# Three zones of cultural competency: surface competency, bias twilight, and the confronting midnight zone

**DOI:** 10.1186/s12909-019-1746-0

**Published:** 2019-08-13

**Authors:** Tanisha Jowsey

**Affiliations:** Centre for Medical and Health Sciences Education (CMHSE), University of Auckland, Auckland City Hospital, Building 599, level 12.025, 2 Park Rd. Grafton, Auckland, 1142 New Zealand

**Keywords:** Cultural competence, Education, Teaching, Culture, Assessment, Cultural safety

## Abstract

**Background:**

Regulatory authorities in healthcare are authorised to develop and assess the cultural competence of their professionals. There remains significant diversity on approaches to cultural competency training and assessment. Little evidence exists about whether existing cultural competency training leads to improved patient health outcomes and reductions in health disparity.

**Discussion:**

In this paper I frame cultural competency as analogous to the ocean and consisting of three zones: surface competency zone, bias twilight zone, and the confronting midnight zone. The surface competency zone focuses on deployment of culturally-specific knowledge: what people see, say, and do. The bias twilight zone is where people engage in critical reflection on their inherent/unconscious biases, and how such biases inform their thoughts and practices. The confronting midnight zone is where people engage in critical consciousness and self-awareness. Here they look beyond their biases to interrogate their power and positionality in society (their own privileges and centralisation). This attention is coupled with a commitment to social justice and to working within their means to reduce health disparities.

**Conclusions:**

I suggest surface cultural competency is somewhat easier to see, teach and reach than the bias twilight or confronting midnight zones. But it is these deeper zones that cultural competency training needs to attend to if we are to see systemic cultural changes in healthcare provision. Research assessing the extent by which cultural competency training within each zone informs improved patient outcomes and reductions in health disparity is called for.

## Background

Internationally, cultural competence is currently discursively operationalised as an important aspect of health professions’ curricula and practices of care on the basis that it contributes to reducing ethnic disparities in health care [1–5]. As such, cultural competence features in clinical education; often in terms of clinical competence, cultural safety, and cross-cultural education [6, 7]. The term cultural competence is relatively new, originating from Cross and colleagues in 1989 who proposed improvements to American health services for children of “color” who experienced severe health disparities [8]. Cross et al. explored cultural competence as a continuum (cultural destructiveness > cultural incapacity > cultural blindness > cultural pre-competence > cultural competence > cultural proficiency) and suggested strategies for developing health service professionals’ competence. Anthropologists critical of the term ‘cultural competence’ proposed alternatives such as ‘structural competence’ [9] and ‘cultural humility’ [10, 11], whereby more emphasis lies with the ways in which societal structures inform individual positionality and agency. In education ‘intercultural competence’ has gained traction [12, 13]. While in healthcare sciences, ‘cultural competence’ remains the dominant accepted term [6, 14, 15], it can be conceptualised as part of a continuum towards patient safety: cultural awareness → cultural sensitivity → cultural competence → cultural humility → cultural safety.

Regulatory authorities in healthcare are authorised to develop and assess the competence – including cultural competence – of their professionals [2, 16]. When cultural competency entered medical and health science curricula in the 1990s, it focused largely on interpreter services and increasing people’s knowledge about how people from specific ethnicities approached a particular health issue. Since then training has developed to focus on critical reflection about how biases inform practices of care. Significant diversity in cultural competency training exists [3].

Two key terms operate in healthcare cultural competence discourse; safety and competence [17–19]. Patient safety is paramount. This includes their cultural safety [20]. Durie explains “cultural safety centres on the experiences of the patients, or clients, while cultural competence focuses on the capacity of the health worker to improve health status by integrating culture into the clinical context” ([21]: 2). Nguyen adds, “cultural safety provides a framework for engagement with patients so that patients can assert power and control over their own health and wellbeing” ([22]: 991). Patients deserve equitable access to culturally-safe practices of care, [23] as do their health care professionals [24, 25].

Cultural competence involves people treating people in a way that makes them feel that their ideas, values, traditions, or behaviours are acknowledged and respected. It is what we do to promote cultural safety, equality, and equity. It has been defined as the “capacity to act in order to support culturally and linguistically appropriate services. Embedded in the concept of cultural competence are knowledge, conviction, and capacity for action at an individual and organisational level (Audigier 2000)” ([26]: 6). Table one outlines established key characteristics of cultural safety and competence (Table 1).

Systematic reviews of cultural competency training such as from Price et al. (2005) and Truong et al. (2014) make clear that many approaches to cultural competency exist and relatively few are adequately evaluated [5, 30]. The diversity of approaches speaks to diversity in understandings about cultural competency. In this paper, I propose to add clarity by framing three broad types of cultural competency.

**Fig. 1:**
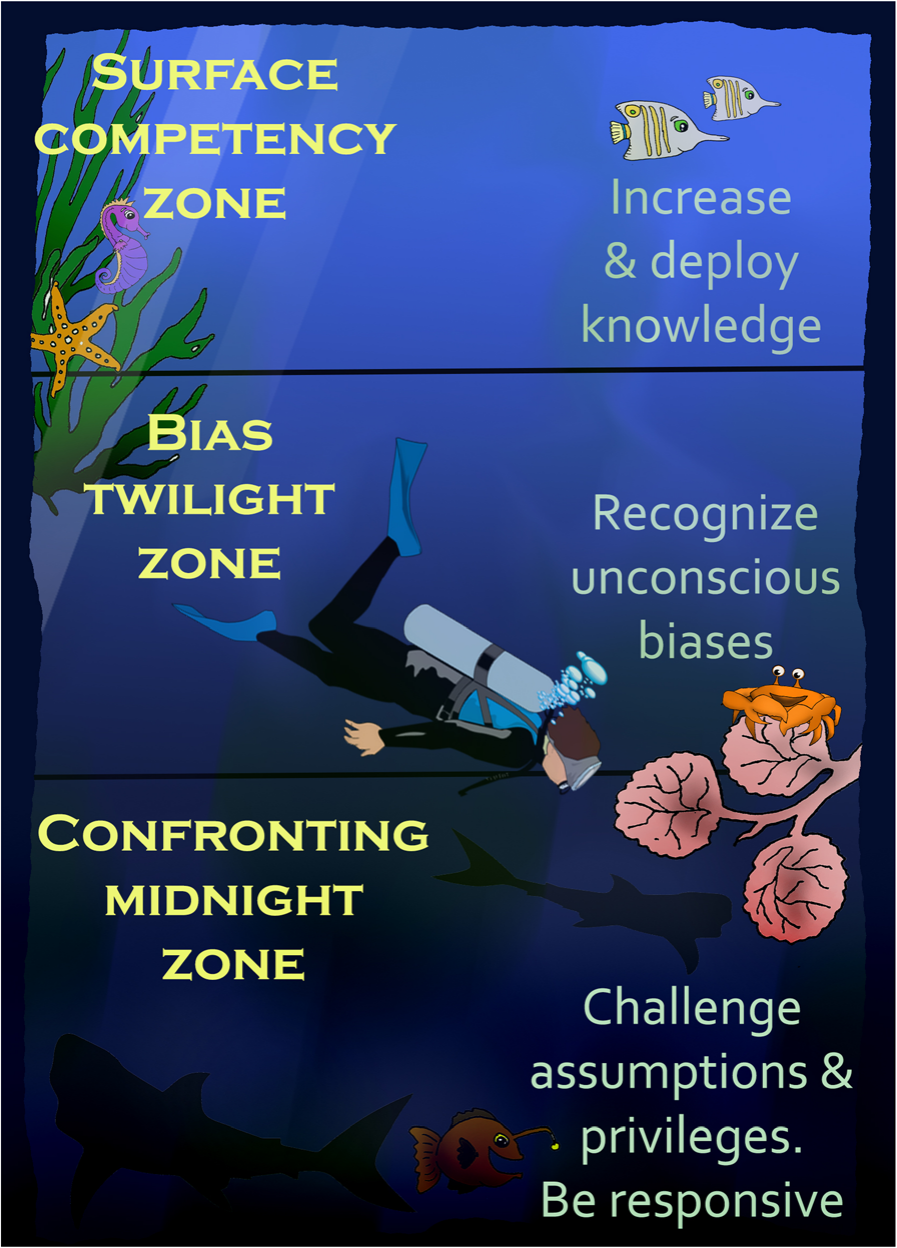
Scuba down through the Competency Zones. Figure detail: This figure contains two elements freely available for reuse with modification 1) Diver PNG – from pngimg.com clipart (res 400 × 308) license Creative Commons 4.0 BY-NC (https://svgsilh.com/image/971329.html), 2) SVG.animal fish ocean aquatic – from SVG SILH license Creative Commons CCO (http://pngimg.com/download/45665), used with thanks. All other elements in the image were created and compiled by Tanisha Jowsey

Oceanographers have mapped ocean depth and light penetration to establish three zones: sunlight (euphotic), twilight (disphotic), and midnight (aphotic) [31]. As depth increases, light penetration decreases. At 1,000 metres the midnight zone begins. I frame cultural competency as analogous to the ocean and consisting of three zones: surface competency zone, bias twilight zone, and the confronting midnight zone. I suggest surface cultural competency is somewhat easier to see, teach, and reach than the bias twilight or confronting midnight zones. But it is these deeper zones that cultural competency training needs to attend to if we are to see systemic cultural changes in healthcare provision toward increased health equality and healthcare equity (see Fig. 1).

**Table 1:** Key established characteristics of cultural safety and competence

Term	Characteristics
Cultural safety	• The person experiences that their culture is respected • The person experiences culturally and linguistically appropriate services • The person is supported to assert control over their own health and wellbeing. They make a decision and have the capacity to act on it
Cultural competence	• Culturally and linguistically appropriate spaces are provided • Culturally and linguistically appropriate services are provided • The person utilises/demonstrates cultural and linguistic knowledge. In healthcare, this means the person promotes effective communication with patient and/or their family • The person utilises/demonstrates conviction of culturally and linguistically diverse knowledge/values/beliefs • The person identifies own biases and how biases could inform the way they treat others; then adjusts thoughts/language/behaviour to minimise influence of their inherent biases on others • The person demonstrates capacity for action to support culturally and linguistically diverse people, and to reduce inequity and inequality. In healthcare, this means the person provides equitable quality of care.

Table 1 details: the characteristics for this table are based on a synthesis of relevant literature [27, 18, 17, 28, 29, 15, 30]

### The surface competency zone

At the surface of the ocean (the first 200 m of water depth) we see huge diversity in marine life; from starfish to coral and angel fish to sharks. The surface competency zone of cultural competence is similar. It hosts diversity in approaches to cultural competence and cultural safety. In this zone focus is on deployment of culturally-specific knowledge: what people see, say, and do. Examples include the provision of culturally-appropriate services such as interpreter services or prayer spaces in hospitals. At an individual level, examples include greeting people in their native language or demonstrating other culturally-specific knowledge. To illustrate, some cultures hold taboos about blood; whether blood should be transfused, shared, or returned to the patient following testing. A person who knows this about a culture with whom their patient identifies might ask their patient whether blood taboos need to be considered in their case. The strength of such an approach – in terms of healthcare education – is that it draws our attention to the multiple ways in which a singular phenomenon can be perceived and addressed.

In Taiwan, clinical teachers identified a lack of cultural competence in students, reflecting insufficient training and performance assessment [32]. They recommended training should include identifying cultural differences and increasing student exposure to “‘small cultures’ [sic] [that] can have an impact on health issues, such as betel nut consumption” ([32]: 210). By ‘small cultures’ the authors are referring to a culture – in this case of betel nut consumption – that involves a small number of people. It contrasts with ‘main stream cultures’ in much the same way that we might differentiate an ethnic minority from a majority [33].

Culturally-specific knowledge such as that of blood taboo or betel nut consumption can be helpful in this zone because it offers clues about what kinds of questions people need to ask others for whom they provide care. The important point here is that people should ask, rather than assume, whether such knowledge is relevant to the individual.

In New Zealand, a national eCALD® training programme is available to healthcare professionals. The emphasis of this training is for effective care of people from culturally and linguistically diverse (CALD) backgrounds who are migrants or refugees to New Zealand from Asian, Middle Eastern, Latin American, and African (MELAA) backgrounds [34]. Training focuses on workplaces and on working with patients and it is offered in both face-to-face workshops and as online learning programmes. The introductory three-hour long online learning programme covers cultural and ethnic awareness, sensitivity, and knowledge, and operates largely in the surface competency zone. Multi-choice questions are used to assess learning. The website hosts these online learning programmes and also links to refugee and migrant services, publications, and screening tools. A similar example from Australia is the national Cultural Competence Program, which is advertised on public television [35]. It draws on a ‘Cultural Atlas’ that profiles cultures from around the world. On the Cultural Atlas home page is the caution:

*Cultural profiles should not be relied upon to form expectations or stereotypes of an individual’s behaviour based on their country of origin. This information is purposed to give you an understanding of the dominant culture of countries so that you may gain insight into the kind of cultural and social environment a first-generation migrant from that country is likely to be familiar with* [36].

This alerts readers to risks associated with the surface competency zone. Critique has emerged concerning the way the term ‘cultural competency’ is used to offer overly simplistic interpretations of culture and perpetuate stereotypes [9, 11, 14, 37–40]. Using cultural knowledge to inform care comes with very real dangers associated with stereotyping cultures. Ramsden writes,

*The idea of a cultural checklist in which heavily stereotyped cultures were able to be predicted by nurses leading to insight on the part of the nurse and conformity and compliance on the part of the patient (Bruni, 1988), was something which I later came to describe as a cultural smorgasbord (Ramsden, 2000). The metaphor was one of ‘cultural tourism’ or ‘voyeurism’, where the nurse stood outside, secure in the culture of nursing, and surveyed the patient from the viewpoint of their interesting exoticism* ([41]: 10).

Ramsden warns that such distancing and stereotyping undermines trust that is essential to competent care [41].

Taylor additionally points out that this educational approach places the learning emphasis on the patient’s culture and virtually ignores the possibility that health care environments, including educational ones, also have cultures that influence and shape the actions of practitioners and patients alike [37]. In Good’s 1993 ethnography of Harvard University medical students, she described the ways in which students learned to position themselves as valid members of medical culture by effectively demonstrating clinical narratives to their teachers and clinicians [42]. They translated patient words into clinically-relevant text for chart notes. In so doing, they established themselves within medical culture, and more importantly, as competent. However, Good observes that this biomedical-centric emphasis on competence left “precious little room for eliciting the kinds of information that might be necessary to establish cultural competence” ([37]: 557).

The surface competency zone is where many of us have started our journeys towards cultural competence. The strengths of this zone include increasing awareness of cultural diversity and deployment of that awareness through effective curious questioning of others. Weaknesses with the surface competency zone include the propensity for simplistic interpretations of people through a cultural lens, distancing and stereotyping.

### The bias twilight zone

Formal cultural competency training is increasingly encouraging trainees to look closely at their own positionality and inherent/unconscious biases. In the twilight zone of the ocean, marine life is harder to see and identify than in the surface zone. Similarly, the bias twilight zone is one in which the individual is encouraged to look at hidden aspects of themselves that are harder to see or perhaps harder to engage with. Literature suggests that educating and supporting people to increase their self-awareness and critical consciousness of biases is more directed towards addressing health disparities than the knowledge/skills/attitudes education associated with the surface competency zone [14, 40, 43].

One path people frequently take to move towards the bias twilight zone is completion of the Harvard Project Implicit Bias Test (https://implicit.harvard.edu/implicit/takeatest.html) followed by group discussion of how biases inform people’s ideas, thoughts, and practices. The bias twilight zone is one in which individuals are encouraged to look more closely at themselves and by so doing increase their awareness of the impact that individuals can have on others. Such awareness can inform their respect for, and responsiveness toward, others [15]. The shift here is quite fundamental as it moves from a universal paradigm back towards an individualistic one.

In 2017, Dao and colleagues in Pennsylvania reported success of their new undergraduate cultural competence program that draws on critical consciousness theory [11]. The training moves away from specific cultural traits and instead embraces reflective strategies - provocation, disorientation, dialogue, heightened consciousness, and re-provocation – to encourage critically conscious approaches to health care. Similarly, Lu and colleagues have suggested cultural competence assessment should include critical reflection [32].

Reflective educational strategies that feature in this zone must be managed with educational sensitivity in order to dissuade learners from seeing cultural competence as a “byword endowed with almost religious significance, a panacea for the multiple and interwoven problems in health care communication” that Perloff and colleagues warn us of ([44]: 835). Essential to successful reflective educational strategies is the learner’s critically conscious engagement with their own biases and ideas about themselves as socio-historically-inflected cultural beings.

In 2018 Kurtz et al. reported a systematic review of cultural safety and competency in Australia, Canada, New Zealand and the United States [3]. Of the 40 reviewed articles, over half “did not report the involvement of Indigenous people in either curriculum development or delivery” ([3]: 274), although the number of articles that did report this increased in recent years. Kurtz et al. suggest that "collaborative partnerships with Indigenous people is key for successful cultural safety program delivery and sustainability. This view discreetly suggests cultural safety is something that is, and should be, targeted towards upskilling non-indigenous people to care for Indigenous people, rather than upskilling people regardless of their specific ethnicity or culture to care for people of diverse ethnicities and cultures. We might caution such a view in light of increasing ethnic and cultural diversity. The Christchurch Mosque Massacre in New Zealand on 15th March 2019 and the Sri Lanka Easter Bombings on 21st April 2019, provide stark illustrations of how biases, racism, and cultural terror move beyond non-indigenous and indigenous paradigms. However, their recommendation speaks back to the ongoing systemic power imbalances maintained through exclusion of Indigenous people, and this is especially relevant as we move deeper into the confronting midnight zone.

### The confronting midnight zone

In this zone, the individual is encouraged to look closely at how their position in society informs their worldview, agency, and power. Tervalon and Murray-Garcia explain that in clinical practice cultural competence “is best defined not by a discrete endpoint but as a commitment and active engagement in a lifelong process that individuals enter into on an ongoing basis with patients, communities, colleagues, and with themselves” ([10]: 118). It is this commitment that people in the confronting midnight zone grapple with. Paul et al. remind us that “addressing health care disparities should be the primary reason for inclusion of cultural competence curricula for health care professionals” ([14]: 753). If we want to realise significant reductions in health disparities then critical consciousness is called for [40]. This is where multiculturalism and social justice connect. Drawing on Freire, Kumagai and Lypson explain:

*Critical consciousness posits that the thinking subject does not exist in isolation but, rather, in relationship to others in the world. The development of critical consciousness involves a reflective awareness of the differences in power and privilege and the inequities that are embedded in social relationships — an act that Freire calls “reading the world” — and the fostering of a reorientation of perspective towards a commitment to social justice. The development of this type of consciousness — a process that Freire calls “conscientization” — is both cognitive and affective and leads to engaged discourse, collaborative problem-solving, and a “rehumanization” of human relationships* ([40]: 783).

For most of us, the personal journey into the confronting midnight zone is not easy. It is confronting and dark. For most non-indigenous non-migrant people, the confrontation stems from decentralisation of self. Di’Angelo explains that for many white non-indigenous people, a commitment to critical consciousness and social justice entails taking a close look at – and ‘sitting with’ the reality of – their own positionality as individuals for whom entire western social systems are geared towards centring and supporting [45]. The flipside of this being that people of other ethnicities and cultures experience the same systems as decentralising and unsupportive, which reduces their experiences of cultural safety, equality and equity - and this is nowhere more stark than in the case of Indigenous people in colonised countries.

DiAngelo [46] challenges normative writings concerning culture by problematizing whiteness in terms of what she calls ‘white fragility.’ White fragility is a state of fragility embodied by white people who have had insufficient opportunity to gain skills for looking closely at their privileged positions, so that when a challenge to that privilege presents itself (usually through a conversation) the white individual feels enormous threat to selfhood and identity, such that they become angry, retaliate or withdraw. The ability of white people to look closely at this positionality in society is further restricted, DiAngelo warns, by the deeply entrenched structures of both universalism and individualism that govern today’s Western societies. She writes,

*Individualism also allows whites to distance themselves from the actions of their racial group and demand to be granted the benefit of the doubt, as individuals, in all cases. A corollary to this unracialized identity is the ability to recognise Whiteness as something that is significant and that operates in society, but to not see how it relates to one’s own life. In this form a white person recognises Whiteness as real, but as the individual problem of other “bad” white people* ([46]: 59).

This deep-seated white-centred structural influence on cultural safety, equality and equity cannot be understated, nor can it easily be undone. The midnight zone of cultural competency is especially confronting for non-indigenous and non-migrant people because it is a zone in which they acknowledge and address the truths in DiAngelo’s words concerning their innate biases, assumptions, beliefs, privileges, and positions in society that inform their agency, actions, and practices of care, as well as their relative inexperience with being racially and systemically attacked for the colour of their skin. The next step is for the individual to commit to doing what they can – as an individual in a position of power – to reduce health disparities.

Pedagogical literature has suggested that one practical way for acting on this commitment is for people to engage in conversations that speak to – and across – cultural difference. This sounds simple enough. But it is not. Jones asks “what if “togetherness” and dialogue-across-difference fail to hold compelling positive meaning for subordinate ethnic groups? What if the “other” fails to find interesting the idea of their empathic understanding of the powerful …?” ([4]: 299). Jones, a non-indigenous white Pākehā academic describes a university course that she co-facilitated with Kuni Jenkins, an indigenous Māori academic. Jones explains that despite their best efforts to create a multicultural learning environment where dialogue-across-difference could thrive, Māori students said “the words, assumptions, and interests of the Pākehā students and lecturer continued to dominate …” ([4]: 300). The teachers therefore separated the students into a Māori/Pacifica group and a Pākehā group and taught the same course material to the two separate groups. Māori /Pacifica students reported feeling “validated”, “vindicated”, and “moved towards the centre.” Whereas Pākehā students were angry and disappointed about the segregation and felt they had missed out on important cultural learning opportunities and “coming together.” Jones’ account clearly demonstrates the generalisability of Di’Angelo’s assertions about white fragility and decentralisation. The point Jones makes is not one concerned with segregation. Rather the focus is on the importance of people creating environments in which voices can be heard. “Most important in educational dialogue” writes Jones, “is not the *speaking voice,* but the *voice heard*” ([4]: 307). For the person committed to engaging with others in the confronting midnight zone the desire is to make things right, to have a conversation, to demonstrate value and respect and responsiveness. But the same person must sit with the confronting reality that they are not central to the conversation, that the conversation may be anything but reassuring to them, and that it is not their voice that needs to be heard.

### Assessment directions

I have proposed here three zones of cultural competency and I suggest that most of the existing cultural competency programmes in place in universities and medical schools teach knowledge and skills associated with the surface competency zone. Few teach into the twilight bias zone, and fewer still into the confronting midnight zone. To respond effectively to situations that call for cultural competency across these zones we – as individuals and as part of broader institutions – need to think carefully – using structured and objective processes – to inform our decisions. Teaching should incorporate and celebrate cultural diversity because doing so promotes cultural inclusion and increases people’s sense of being valued. This may be as simple as teachers focusing on the knowledge side of cultural competence by increasing learner exposure to small cultures or to culturally-specific and relevant knowledge (such as an Indigenous-specific teaching aid for diabetes management) [7]. It may include learning activities that focus on deep exploration of learner core values and attitudes. I also recommend that we focus on the strengths that people bring to situations and practise inclusive behaviours that promote voices heard.

Kirkpatrick’s model of training evaluation (reaction, learning, behaviour change, results) suggests that the apex of training evaluation – in this case of cultural competence – should demonstrate results/impact on patient outcomes [5, 47]. Whether or not cultural competency training results in reducing patient health disparities remains to be seen [3, 6]. This likely reflects both the diversity in approaches to cultural competency training and complexity of addressing health disparities in society.

During 2003–2004, about half of the graduate medical education programmes in the United States (8000) offered cultural competence training [48]. Around the same time New Zealand saw various cultural competency interventions initiated in medical education [49] and healthcare systems, including:

*programs to recruit and retain staff members who reflect the cultural diversity of the community served, use of interpreter services or bilingual providers for clients with limited English proficiency, cultural competency training for healthcare providers, use of linguistically and culturally appropriate health education materials, and culturally specific healthcare settings* ([50]: 68).

In 2005, Price and colleagues’ systematic review identified 64 articles evaluating cultural competency training programmes, only four of which measured patient outcomes (but not patient health outcomes) [30]. They additionally note, “only 27 of the 64 studies used objective evaluation strategies (written examinations, direct observation, performance audit, validated self-efficacy scales)” ([30]: 581). Price et al. suggest that even if training evaluations did not show improved patient outcomes, the training could still be valuable beyond learner satisfaction. More recent studies by Lei et al. (2011), Truong et al. (2014), and Kurtz et al. (2018) drew similar conclusions [3, 5, 6].

The Purnell Model for Cultural Competence (1991) draws attention to specific areas of culture such as pregnancy, death rituals, nutrition, and communication, for which specific cultures often have specific values, beliefs, and practices [51]. Jones and Pinto Zipp (2018) have recently developed and validated a survey tool for assessing cultural competence, which draws specifically on the Purnell Model. Their research shows the Global (worldview) Cultural Competence Survey is effective in assessing cultural competency levels in health profession students [52]. The Association of American Medical Colleges similarly offers an assessment checklist that assesses knowledge, skills, and attitudes [53].

Poland has recently seen a significant rise in cultural diversity, requiring increased cultural competency among health professionals [54]. Barzykowski et al. adapted, implemented, and evaluated cultural competency training using the Cross-Cultural Competence Inventory (CCCI) and the Cultural Intelligence Scale. The CCCI includes 63 items under the seven dimensions: cultural adaptability, self-presentation, tolerance of uncertainty, determination, engagement, mission focus, and Lie and Social Desirability Scale. Barzykowski et al. report training and evaluation research with 725 Polish national students (almost all of whom were medical or nursing) was completed. Their research ‘proved’ the theoretical reliability and validity of the CCCI and identified significant positive correlations with the Cultural Intelligence Scale. Two dimensions of the CCCI (cultural adaptability and engagement) vaguely relate to the surface zone of cultural competency that I am proposing, and one dimension (self-preservation) of the CCCI is relevant to the twilight bias zone. While the CCCI fails to engage adequately with the bias twilight or confronting midnight zones, it is heading in the right direction with these dimensions.

I suggest these approaches - The Purnell Model for Cultural Competence, Global (worldview) Cultural Competence Survey, CCCI, and the Cultural Intelligence Scale – are likely to accurately identify surface competency zone skills. However, I am less confident in their capacity to identify people operating in the twilight or midnight zones. What we desperately need now is creative empathetic thinking about how we can implement and assess training that attends to these two deeper darker zones; research that can speak to Kirkpatrick’s model and ascertain the extent by which reduction in health disparities can be realised through cultural competency training. Such training must be coupled with system changes oriented toward reducing structural disparity; as a starting point, curricula should holistically embed decolonization approaches and train and assess for Indigenous health care competencies [55]. Otherwise, our individual efforts risk amounting to mere drops in a very deep ocean.

## Conclusion

This article has traced important historical and current trends in cultural competence training. I have framed cultural competency as consisting of three zones: surface competency zone, bias twilight zone, and the confronting midnight zone. I suggest surface cultural competency is somewhat easier to see, teach, assess, and reach than the bias twilight or confronting midnight zones. But it is these deeper zones that cultural competency training needs to attend to - in concert with structural changes to medical curricula - if we are to see systemic cultural changes in healthcare provision and reductions in health disparities.

## Data Availability

Not applicable.

## Abbreviations

CALD: Culturally and linguistically diverse
CCCI: Cross-Cultural Competence Inventory
MELAA: Asian, Middle Eastern, Latin American, and African
